# Designing and evaluating contextualized drug–drug interaction algorithms

**DOI:** 10.1093/jamiaopen/ooab023

**Published:** 2021-03-19

**Authors:** Eric Chou, Richard D Boyce, Baran Balkan, Vignesh Subbian, Andrew Romero, Philip D Hansten, John R Horn, Sheila Gephart, Daniel C Malone

**Affiliations:** 1 Department of Biomedical Informatics, University of Pittsburgh, Pittsburgh, Pennsylvania, USA; 2 College of Engineering, University of Arizona, Tucson, Arizona, USA; 3 Banner University Medical Center, Tucson, Arizona, USA; 4 Department of Pharmacy, University of Washington, Seattle, Washington, USA; 5 College of Nursing, University of Arizona, Tucson, Arizona, USA; 6 Department of Pharmacotherapy, University of Utah, Salt Lake City, Utah, USA

**Keywords:** decision support systems, clinical, drug interaction, knowledge bases

## Abstract

**Objective:**

Alert fatigue is a common issue with off-the-shelf clinical decision support. Most warnings for drug–drug interactions (DDIs) are overridden or ignored, likely because they lack relevance to the patient’s clinical situation. Existing alerting systems for DDIs are often simplistic in nature or do not take the specific patient context into consideration, leading to overly sensitive alerts. The objective of this study is to develop, validate, and test DDI alert algorithms that take advantage of patient context available in electronic health records (EHRs) data.

**Methods:**

Data on the rate at which DDI alerts were triggered but for which no action was taken over a 3-month period (override rates) from a single tertiary care facility were used to identify DDIs that were considered a high-priority for contextualized alerting. A panel of DDI experts developed algorithms that incorporate drug and patient characteristics that affect the relevance of such warnings. The algorithms were then implemented as computable artifacts, validated using a synthetic health records data, and tested over retrospective data from a single urban hospital.

**Results:**

Algorithms and computable knowledge artifacts were developed and validated for a total of 8 high priority DDIs. Testing on retrospective real-world data showed the potential for the algorithms to reduce alerts that interrupt clinician workflow by more than 50%. Two algorithms (citalopram/QT interval prolonging agents, and fluconazole/opioid) showed potential to filter nearly all interruptive alerts for these combinations.

**Conclusion:**

The 8 DDI algorithms are a step toward addressing a critical need for DDI alerts that are more specific to patient context than current commercial alerting systems. Data commonly available in EHRs can improve DDI alert specificity.

## BACKGROUND AND SIGNIFICANCE

Drug–drug interactions (DDIs) are associated with an elevated risk of hospitalization in older adults and are responsible for an estimated 5%–14% of adverse events among inpatients.^1^^,^^2^ While failure to properly manage a DDI is a medical error,^3–5^ prescribers and pharmacists often have inadequate knowledge regarding DDIs and how to properly manage an interaction when patient exposure cannot be avoided.^6–10^ Electronic prescribing and pharmacy systems include alerts for potential DDIs as a form of clinical decision support (CDS) to warn prescribers and pharmacists of potentially harmful medication combinations, and ideally provide documentation on how to avoid or mitigate the risk of patient harm. However, this technology has led to unintended consequences and fallen short of fulfilling its potential to improve patient safety.

Clinicians override (ie, ignore or dismiss warning) for up to 90% of potential DDI alerts, primarily because clinicians do not consider the alerts to be relevant.^11–15^ Wright et al^14^ examined DDI alert override reasons across 10 sites and found “not clinically significant” to be the second most commonly provided reason that clinicians provided for overriding alerts. Excessive irrelevant alerts are thought to decrease users’ sensitivity to alerts, producing what is known as the *cry-wolf phenomenon*, *alarm fatigue*, or more specifically *alert fatigue.*^10^^,^^13^^,^^14^ Alert fatigue is more than just a frustration; it can lead clinicians to respond inappropriately.^15^ Edrees et al^16^ found that 87.3% of high priority alerts were overridden in a 1-year sample of inpatient and outpatient data from a large academic health system. Of concern, less than half (45.4%) of the overrides were considered appropriate. Moreover, the rate of adverse drug events was higher with inappropriate versus appropriate overrides (9.4% vs 4.3%; *P* = .038).

Approaches to reduce alert fatigue include turning-off entire categories of alerts, using expert opinion to refine commercial drug knowledge bases to a smaller set of potential DDIs, and tiering alerts by relative clinical importance.^11^^,^^12^^,^^17–19^ For example, Bakker et al^20^ conducted a modified Delphi study to identify DDI alerts that are of importance to critical care patients and found that 38% of 148 potential DDIs were not clinically relevant for the ICU. Pirnejad et al^21^ used clinical input from a nephrology team to redesign potential DDI alerts for kidney transplant patients and reduced the number of DDI alert types from 52 to 33. A 2020 consensus paper by 4 large health care institutions in the United States describes additional actions such as using an appropriate alert governance and management process, collecting and monitoring alert performance metrics, and improving alert presentation.^22^

Unfortunately, most systems currently trigger DDI alerts based on the pair-wise combinations of the drugs involved. Thus, there tends to be little or no consideration by the systems of contextual factors. However, the specificity of an alert to individual patient characteristics play a major role in alert acceptance.^11^^,^^23–25^ An *in situ* qualitative study on prescribers’ interaction with electronic medication alerts showed that when alerts failed to provide contextual information, prescribers bypassed the alert and then searched for the relevant data that they needed.^26^

Quality improvement projects have found that making DDI alerts more appropriate to clinical context can improve alert acceptance. Daniels et al^15^ observed a reduction in the override rate from 93.9% to 46.8% after making nearly a third (30.2%) of DDI alerts more contextual and suppressing another 16.5% of alerts. Similarly, Muylle et al^27^ were able to reduce alerts that interrupt clinician workflow (interruptive alerting) for potassium increasing potential DDIs by 92.8%, with no statistically significant effect on the rate of hyperkalemia, by restricting alerts to only cases where a recent potassium value was ≥5 mMol/L.

### Objectives

The primary audience for this work is informatics leaders at health systems who are responsible for and/or interested in implementation and evaluation of CDS systems. The overarching goal of our work is to develop and validate algorithms that use data available in electronic health records (EHRs) as contextual information to provide greater specificity to DDI alerts that are frequently overridden. In this study, we describe the creation of algorithms that were validated on both synthetic and real-world EHR data. The algorithms apply to adult patients exposed to 1 or more of the interacting drug combinations. We expect the DDI algorithms to have significant impact because the involved drug combinations account for a disproportionate share of alert overrides.^15^^,^^21^ Moreover, we provide both logic flow diagrams and computable artifacts the DDI algorithms that should help others to adapt them to their setting.

## METHODS

### Identification of high priority DDIs and algorithm development

High priority DDIs were identified based on the most frequently overridden alerts at the former “University of Arizona Medical Center” (now “Banner University Medical Center Tucson”) over a 3-month period (Table 1A and B). A necessary condition for the DDIs to be “high priority” for this project was that more than 1000 overrides were observed. Drug experts on the research team (4 pharmacists trained in DDIs: AVR, DCM, JRH, and PDH) also assessed if evidence for potential harm existed in the literature, if the alert lacked specificity as implemented in the health system, and if the alert would be amenable to contextualization using EHR data, such as laboratory values, diagnoses, duration of use, and other factors that would affect the risk of harm. The experts then performed an extensive literature review to identify drug-related and patient-related factors that would impact the risk of harm from exposure to each interacting drug combination (Table 1C). The drug experts used the results of the literature review to design DDI alert algorithms that were contextualized to risk increasing or mitigating factors. The team discussed each algorithm as it was developed over regularly scheduled web meetings.

**Table 1.** Overview of the study activities arranged to distinguish steps that were conducted to design contextualized drug–drug interaction algorithms (A–D) and steps conducted to test the rules on both simulated and real-world electronic health records data (E–I)

Study activity		Lead personnel
Algorithm design		
A	Data collection at the University of Arizona Medical Center	DCM and AVR
B	Identification of highest number of alert overrides based on data	DCM and AVR
C	Literature search to develop clinical algorithms	JRH and PDH
D	Manual clinician review of random patient profiles for each algorithm	DCM
Algorithm testing		
E	Validation of rule output via relational database queries	EC
F	Source real-world EHR data loaded in the Common Data Model and run through rules	SCR and EC
G	Testing of rules using a simulated population loaded in the OHDSI Common Data Model	RDB, SCR, and EC
H	Translation of clinical algorithms into Drools rules	RDB, SCR, and EC
I	Development of standardized value sets for drugs, conditions, and labs in clinical algorithms	DCM, RDB, and SCR

**Figure 1.**
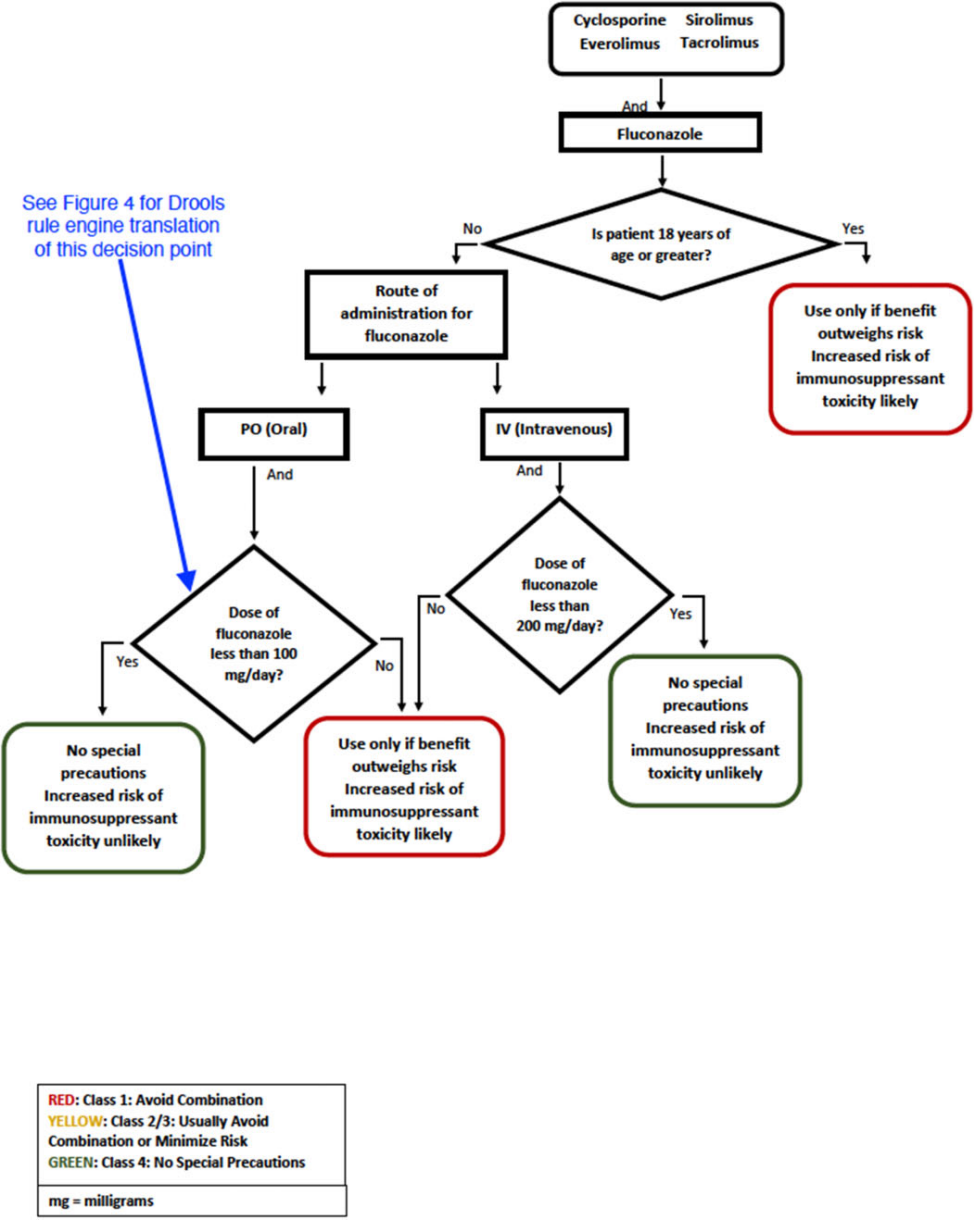
The algorithm for “Immunosuppressant/Fluconazole” drug–drug interaction decision support as a document-based decision tree.

Each algorithm was developed as document-based decision tree (also known as a logic flow diagram, see an example in Figure 1) specific to the potentially interacting drug pair. The leaf nodes of each DDI algorithm decision tree indicated the seriousness of the potential DDI and potential operational recommendations (see Table 2). The drug experts documented supporting evidence for every branch point in the DDI algorithm. Additionally, the team wrote protocols to define terminology code sets for the relevant medications, conditions, and lab measurements for each rule. These protocols were used to create extensional concept sets for the 8 algorithms.

**Table 2.** Operational classifications assigned to the leaf nodes of the potential DDI algorithms

Color coding	General clinical recommendation
Red	Avoid Combination
Yellow	Usually Avoid Combination or Minimize Risk
Green	No Special Precautions

### Implementation of computable algorithms and testing against simulated data

Members of the research team then translated the document-based decision tree algorithms into knowledge artifacts implemented rules using JBoss Drools (Table 1H). The rules were written so that a rule engine could identify patients that satisfy specified criteria for each decision in the DDI decision tree. Supplementary Figure S1 shows an example Drools rule corresponding to the potential DDI decision tree in Figure 1.

For the purpose of creating a sample implementation, and to test that the rules would run as expected, a synthetic patient population was created and loaded into the open source common data model provided by the Observational Health Data Science and Informatics (OHDSI) collaborative.^28^ This common data model was chosen for its ability to accommodate all of the different types of observational health data used by the algorithms as well as its use of widely-used standardized terminologies. These features made it simple to use concept sets to refer to the medications, observations, conditions, and measurements used in the DDI algorithms. The knowledge artifacts for each DDI algorithm were executed against simulated patient data (Table 1G) to validate that the algorithm’s logic was sound and complete.

The DDI rules were designed to use concept sets to refer to clinical entities such as drug exposures, conditions, and measurements used in the original algorithms. Concept sets were created for the entities using terms from RxNorm (drugs), LOINC (lab measurements), and SNOMED-CT (clinical conditions) terminologies (Table 1E). For each entity, the team identified candidate concepts from the relevant terminology in a systematic manner, starting with locating a more general concept and then assembling a list of descendant concepts. These were then iteratively filtered by the team to remove nonrelevant concepts. The final concept sets were loaded into a database table used by the rule engine. They were also published to the National Library of Medicine’s Value Set Authority Center.^29^ The final set of rules and concept sets were used to help with quality assurance alongside queries of the relational database (Table 1I).

### Application of computable algorithms to real world data

**Figure 2.**
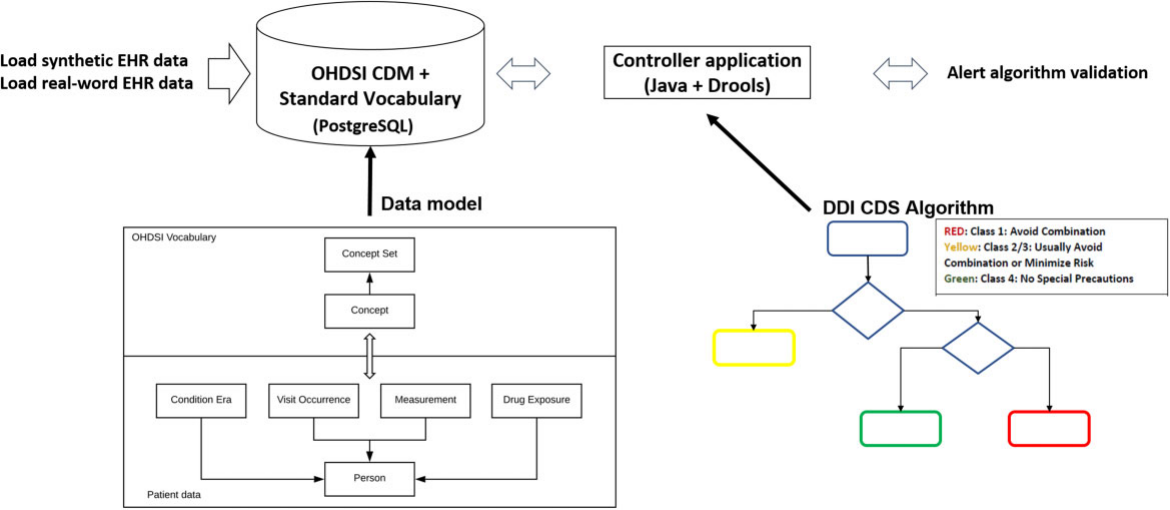
A flow diagram showing the key programming activities and software artifacts for the study. The study team loaded simulated and real-word EHR data into separate schemas within a single database instance (PostgreSQL). Both database schemas using the common data model and standard vocabulary maintained by the OHDSI collaborative. All data were person-centric and coded using terminology standards stored in the Concept and Concept Set tables. The research team used a custom Java-based controller to load data from the database into Drools working memory and run the computable potential drug–drug interaction alert algorithms for testing and validation. DDI: drug–drug interaction; CDS: clinical decision support.

Figure 2 shows a flow diagram showing the key programming activities and software artifacts for the study. The rules were also evaluated using real-world data to identify the feasibility of translating them “outside of the laboratory” into real clinical workflows. After obtaining Institutional Review Board (IRB) approval, 3 months of de-identified data were obtained from the University of Arizona Medical Center (January 1, 2016 to March 31, 2016). Data were extracted only for those patients who had at least 1 exposure to a drug that was included in 1 of the 8 DDI algorithms. These source data files included records for patient diagnoses, drug orders, and lab results, which served as entities to describe a population to be used for evaluating DDI algorithms. The research team transformed the data into the OHDSI common data model, which allowed reuse of the rules that were validated using simulated data with only minor modifications (Table 1H).

The simulated patient data and the real world population data both contained similar types of observational data related to medications, conditions, lab measurements, and the patients’ visits to the facility (Figure 2). Thus, both datasets could be loaded following similar OHDSI common data model schemas using the PostgreSQL database management system. Additionally, the robust support for different coding systems in the common data model allowed for seamless mapping of the full breadth of data in both the simulated and real world datasets to the curated concept sets. The Drools rule engine could connect to either datasets interchangeably by configuring an application programming interface (Java Database Connectivity) Driver that linked the rule engine to the PostgreSQL database.

DDI algorithm execution over real-word data was validated against SQL queries of the relational database with patient population data (Table 1I). These queries were written independent of the rule engine so that daily alert counts per rule could be compared. Comparisons of the query output with that of the rule engine informed further refinements of the rule engine. Each rule was considered validated for use with the real world data when the alert and query output were concordant. The research team defined basic concomitant exposure to the 2 drugs of interest for each DDI as a simple overlapping exposure period (meaning that the start and stop dates of the 2 drugs overlapped). Alongside this output, we analyzed demographic attributes of the patients and the availability of drug and patient risk factors that were included in the clinical algorithms for each of the rules.

We counted 2 types of output: basic concomitant exposure to the 2 potentially interacting drugs in each algorithm, and occurrences of the various rule algorithm leaf nodes labeled using the codes shown in Table 2. Every unique exposure to the potentially interacting drugs indicated by an algorithm was counted. Cases where multiple drug exposures were started for the same drug at the exact same timestamp, but with different directions for dosage, were counted separately in the output. Supplementary Figure S2 shows 3 example scenarios where the same patient would have multiple alerts on a given day involving the same potentially interacting drug pair.

## RESULTS

### Identification of high priority DDIs and algorithm development

A total of 8 DDIs were identified as high priority for contextual alerting. These are shown in Table 3 along with the drug and patient factors that were used in the contextualized potential DDI algorithms. The document-based decision tree for each algorithm is provided in Supplementary Appendix with a sub-set of algorithms published to https://ddi-cds.org/. Both document-based decision trees and computable knowledge artifacts are publicly available for a subset of algorithms at https://ddi-cds.org. The source code used to implement the Drools knowledge artifacts is also available on the web.^30^ Moreover, a Drools environment containing the rules, rule execution environment, and synthetic data are available as a Docker image.^31^

**Table 3.** The 8 DDIs that were selected for development of contextualized decision support algorithms

Drug–drug Interaction	Drug Factors	Patient Factors
Citalopram/QT prolonging agent	Dose of citalopram QT-agents	Female sex, ECG > 480 ms, age 68 or older, concomitant loop diuretic, serum potassium < 3.5 mEq, history of myocardial infarction, has diagnosis of sepsis, and has diagnosis of heart failure
Clonidine/beta-blocker	Class of beta-blocker (selective, nonselective, alpha-blocking) Clonidine formulation Timolol formulation	Withdrawal of clonidine
Epinephrine/beta-blocker	Class of beta-blocker (selective, nonselective, alpha-blocking) Epinephrine formulation Timolol formulation Epinephrine indication (dermatological, dental, plastic surgery uses) Dose of epinephrine	Anaphylaxis conditions
Fluconazole/opioid	Dose of fluconazole Dose of oxycodone Dose of fentanyl	Visit type (inpatient or outpatient)
Immunosuppressant/fluconazole	Immunosuppressants Fluconazole formulation Dose of fluconazole	Age
Potassium/potassium-sparing diuretic	Potassium Dose of spironolactone Dose of amiloride Dose of triamterene	Serum potassium concentration
Warfarin/antidepressant	Warfarin SSRIs and SNRIs Tricyclics Buproprion Mirtazapine NSAIDs Aspirin Systemic corticosteroids Aldosterone antagonists Anti-platelet medications	Age History of UGIB or peptic ulcer
Warfarin/salicylate	Warfarin Salicylate formulation Nonacetylated salicylates Dosage of nonacetylated salicylates	Indication of thromboembolic events

*Note*: The column to the right shows the health record data types that were used to contextualize the algorithms. The table also shows health record data types that were used to make the algorithms specific to specific patient situations.

*Abbreviations:* K: potassium; NSAIDs: nonsteroidal anti-inflammatory drugs; QT: QT interval; SNRIs: serotonin-norepinephrine reuptake inhibitors; SSRIs: selective serotonin reuptake inhibitors; UGIB: upper gastrointestinal bleeding.

### Implementation of computable algorithms and testing against simulated data

Eight computable DDI algorithms were successfully validated using Drools and simulated patient data. The synthetic patient population included 93 people with a total of 24 lab measurements, 12 condition occurrences, and 208 drug exposures. This population, though small, was sufficient to validate that Drools rules identified all patients qualifying for each branch of logic in each algorithm and did not trigger incorrect decisions for any patients. Almost all clinical entities required for the DDI rules were supported in the version 5 of the OHDSI common data model. Only 1 algorithm had a decision tree branch that was not possible to implement in the computable version. Specifically, the “Epinephrine/Beta-blocker” algorithm referenced the use of epinephrine for dermatology, dentistry, or plastic surgery. This could not be implemented in the computable rule because the OHDSI common data model does not directly link drug exposures with patient condition occurrences.

### Application of computable algorithms to real-world data

The real-world EHR dataset included 24 599 individual patients who had a healthcare encounter that overlapped with the study’s 3-month period (January–March 2016). The average age of all patients at the end of the study period was 43.4 (SD = 23.7) years of age. There were 31 332 distinct health encounters with 10 506 (33.5%) having a duration of at least 24 hours. The manner in which the data were extracted did not allow us to identify the type of healthcare encounter each patient had (inpatient, emergency department, or outpatient). So, we focused the analysis on encounters lasting 24 hours or more. Tables 4 and 5 summarize the clinical dataset from real-world EHRs used in this study.

**Table 4.** Demographics of the real-world electronic health records that the DDI algorithms were tested on

Patient characteristics	*N* (%)
Total	24 599
Sex	
Female	13 164 (53.5)
Male	11 435 (46.5)
Ethnicity	
Hispanic or Latino	10 407 (42.3)
Not Hispanic or Latino	13 748 (55.9)
Unknown	444 (1.8)
Age	
Under 18 years old	3843 (15.6)
18–60 years old	13 682 (55.7)
60+ years old	7074 (28.7)

**Table 5.** Summary information on the real-world electronic health records dataset that the DDI rules were tested on

	*N*	Average per person (SD)
Any health encounter		
Number of visits	31 332	1.27 (1.13)
Visit length		2 d, 5 h, 18 min
Drug exposures started during visit	516 387	17.3 (29.0)
Lab measurements taken during visit	284 657	16.8 (31.5)
Health encounter of ≥24 h		
Number of visits	10 506	1.14 (0.46)
Visit length		6 d, 3 h, 10 min
Drug exposures started during visit	336 789	32.3 (43.2)
Lab measurements taken during visit	234 353	27.8 (41.8)
Visit duration of <24 h		
Number of visits	20 826	1.22 (1.18)

*Note:* All drug exposures and measurements considered in this table are counted if and only if they are recorded during a visit within the study period. A visit was determined as occurring within the study period if it either began or ended between the 3-month period of January through March 2016.

**Figure 3.**
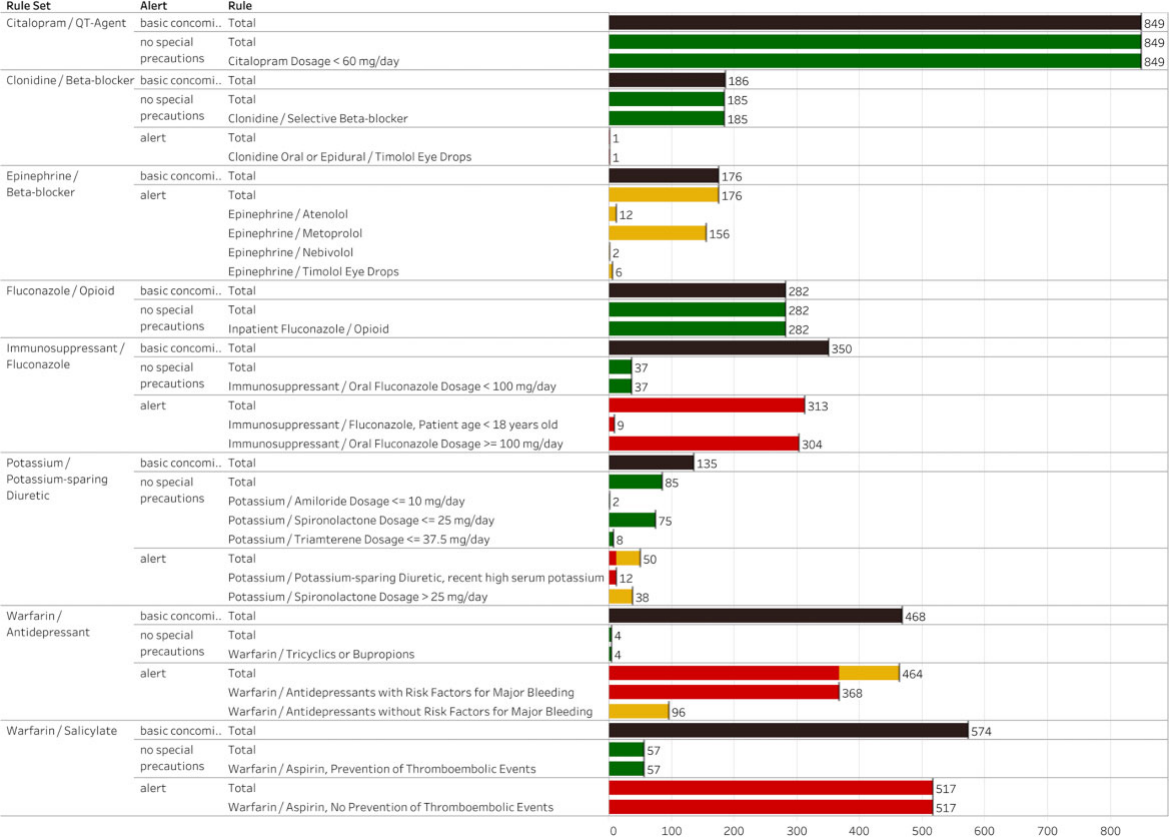
The results of running the 8 computable algorithms for which there was concomitant exposure in the real world dataset. The figure shows a set of 2 bar charts for each algorithm. The first bar chart shown for each algorithm shows the total count of basic concomitant exposure to the drugs involved in the algorithm DDI. The second bar chart shown for each algorithm shows 2 counts derived from the simulated alerts triggered by the computable algorithm. The first count is the total number of simulated alerts that triggered by each rule. The second count is a breakdown of the specific branches of each algorithm that were triggered. Color coding is used to indicate the operational classification of each branch based on Table 2 (red = Avoid Combination; yellow = Usually Avoid Combination or Minimize Risk; and green = No Special Precautions).

Figure 3 shows the results of testing the computable rules for the 8 algorithms over the retrospective real-world dataset. All the DDI algorithms were based on drug combinations that frequently triggered alerts at the study site. Thus, if one assumes that in a typical EHR, basic concomitant exposure to drugs involved in the selected DDIs will always result in an *interruptive* alert (ie, a modal dialogue box interrupting the ordering clinician’s workflow), then the total number of alerts that would have triggered based on basic concomitant exposure (black bars in Figure 3) is 3020. Figure 3 shows that the 8 algorithms suggest filtering 1584 (52.4%) of these alerts based on the operational classification “No Special Precautions” (green bars in Figure 3). Examining specific DDIs, the percentage of interruptive alerts that the algorithms suggest completely filtering (“No Special Precautions”) ranges from 100% for “Citalopram/QT prolonging agent” (*N* = 849) and “Fluconazole/Opioid” (*N* = 282) to <1% for Warfarin/Antidepressant (*N* = 468).

The algorithm that resulted in the most contextualized simulated alerts was “Warfarin/Antidepressants.” In contrast to 468 basic concomitant exposures with no contextualization, the computable rule identified 368 (78.6%) situations classified as “Avoid Combination,” 96 (20.5%) situations classified as “Usually Avoid Combination or Minimize Risk,” and 4 (0.9%) situations classified as “No Special Precautions.” The “Immunosuppressant/Fluconazole” algorithm also resulted in well contextualized output with 313 (89.4%) of the 350 basic concomitant exposures classified into 2 different situations warranting an “Avoid Combination” classification, and 37 (10.6%) classified as “No Special Precautions.” The “Epinephrine/Beta-blocker” algorithm was the only algorithm that transitioned all basic concomitant exposures (*N* = 176) to “Usually Avoid Combination or Minimize Risk.”

## DISCUSSION

Avoiding alert fatigue is critical if warnings are to be useful to clinicians. This study shows proof-of-concept and the value for contextualizing potential DDI alerts using data commonly available in EHRs. Document-based decision trees were developed for 8 potential DDI alerts that occurred frequently at a tertiary care hospital (Supplementary Material). It is notable that an evaluation of the rules on retrospective real-world data from the same institution showed the potential to reduce interruptive alerts by more than 50%. We expect that this level of alert reduction combined with the greater contextualization of interruptive alerts would help reduce alert fatigue among prescribing clinicians working with inpatient adults.

The meaningful use regulation has resulted in health systems with EHRs to also implement DDI warnings based on third-party drug databases.^32^ The developers of these databases recognize that overalerting is a major complaint of their systems, but are also concerned about failing to provide a warning when one should be provided. The current systems are largely built on simple lookup rules that examine the drug ingredient without considering other attributes of the product such as dose, route of administration, timing, and other patient-specific factors that directly affect the relevance of the warning. Furthermore, many of these decision rules lack specificity at the ingredient level, relying on sometimes broad therapeutic categories to assign interaction properties.

Our study seeks to advance DDI algorithms to use contextual factors that improve the specificity of warnings, while retaining a high degree of sensitivity. It is important to stress that, while necessary for reducing DDI alerts and making them more clinically relevant, the approach might not be sufficient to increase alert acceptance or improve clinical outcomes. For example, Duke and Bolchini^33^ designed a model to integrate patient-specific data into DDI alerts via a web-based service that was interoperable across clinical information systems. In a randomized controlled follow-up study, Duke et al^34^ found no improvement in prescriber adherence to DDI alerts that included the patient’s most recent relevant laboratory values for hyperkalemia-associated DDIs among high-risk patients. It is important to note that Duke et al’s alerting system did not use patient-specific data to intelligently filter alerts for clinicians. Rather, the alerts simply showed the relevant laboratory values, assuming clinicians would make the necessary cognitive connections. The authors acknowledged that stating the elevated risk for each patient might have improved adherence, suggesting the importance of addressing alert specificity. For example, alerts for lower-risk patients could have been filtered or downgraded. Such tiering, or prioritizing, of alerts has been shown to improve adherence.^17^ The Duke et al study is not the only one to report no benefit from contextualized DDI alerts. A cluster-randomized controlled study by Beeler et al^35^ of contextualized potassium increasing DDI alerts found that negligible impact on alert management. We note that every alert in the study was noninterruptive, regardless of priority, making it feasible that clinicians missed clinically important alerts even in the intervention group.

Our findings are concordant with the results of other studies that have demonstrated the potential for substantial reductions in alert volume when greater attention is given to contextual factors. Riedmann et al^36^ used a combination of literature searches and expert interviews to design a context model of 20 factors that could be used to prioritize drug safety alerts depending on the characteristics of the specific patient (eg, clinical status), alert (eg, severity), and user or organizational unit (eg, professional experience of user). Seidling et al^37^ observed that more than half of the alerts that would have been triggered for DDIs involving cholesterol-lowering statin drugs were inappropriate because the dose of the statin was not considered by the software. More recently, Seidling et al^23^ identified 14 types of modulators useful for refining the specificity of DDI alerts. These could be applied to 83 out of 100 frequently triggered DDI alerts using relevant factors in the EHR. However, the clinical relevance of this work was limited by the fact that the investigators did not conduct an evaluation of the alerts to determine appropriateness. Horn et al^19^ were able to reduce the potential number of major alerts by nearly 70% by applying an operational classification that specified DDI clinical management strategies to a commercial KB.

Some potential limitations of this study include that the DDI alert algorithms were developed based on potential DDIs that were frequently overridden at a single institution, were potentially harmful to some patients, and were amenable to contextualization. This criterion was applied to data from a single site and by a single group of experts. Other panels might have selected different DDIs based on their institutional and/or clinical context. The real-world data did not indicate the type of health encounter each patient had so we limited the analysis to encounters lasting 24 hours or more. Work is underway to test the DDI algorithms using real-world data from outpatient setting where it can be more challenging to identify a complete set of truly overlapping drug exposures. The algorithms were developed by consensus among a team of leading DDI experts but there has not been any empirical validation that the algorithms are effective at reducing alert fatigue. The computable artifacts were tested on retrospective real-world data using Java-based Drools rules and the OHDSI common data model. Further testing would be needed before implementing the rules in a clinical setting because of differences in EHR and how they are implemented.

As is mentioned above, the primary audience for this work is informatics leaders at health systems who are responsible for and/or interested in implementation and evaluation of CDS systems. However, it is often the case that informatics leaders at healthcare organizations have little or no control over the DDI rule logic used by commercial drug knowledge base vendors with which an organization might contract for DDI CDS. For this reason, we think that commercial systems should seek to work with the major EHR vendors and the standards community (eg, HL7 FHIR) to provide expanded interoperability and allow more contextualized DDI alerts. Indeed, progress has been made in that direction through the process of developing an implementation guide for DDI CDS using emerging health information technology standards.^38^ Multiple rules developed for this project have been implemented using HL7 Clinical Quality Language (CQL) and FHIR using the suggested Minimum Representation of Potential Drug–Drug Interaction Knowledge and Evidence.^39–41^ One of these rules has been used as a use case in the recently balloted implementation guide which has received significant input from EHR vendors, drug knowledge base vendors, regulatory agencies, and other stakeholders.^42^

Another limitation that should be considered is that the interactions of interest in this study are largely for adult populations. While our original intent was not to exclude interactions that may occur in pediatric populations, we focused this study on those interactions with a high override rate at a major medical center. This study did not attempt to quantify the degree of harm prevented because of challenges in determining the frequency that patients are harmed from exposure to a DDI. Another limitation of the study is that designing alerting algorithms is challenging due to the lack of studies on DDIs that consider risk factors directly. Thus, extensive and perhaps more comprehensive algorithm development is limited by lack of evidence.

## CONCLUSION

This work addresses a critical need for contextual DDI algorithms that are well-tested and shareable. The comprehensive evaluation of the 8 algorithms for DDI frequently overridden alerts serves as an important step towards making DDI alerting more patient specific across various healthcare organizations.

## SUPPLEMENTARY MATERIAL

Supplementary material is available at *Journal of the American Medical Informatics Association* online.

## AUTHOR CONTRIBUTIONS

All co-authors made substantial contributions to the design of the work and the analysis and interpretation of data. DCM and AR acquired the electronic health records data for the work. JRH, AR, DCM, and PDH developed the drug–drug interaction clinical decision support algorithms. EC, RDB, BB, and VS developed the knowledge artifacts and conducted the primary analysis. SG provided input on the algorithms and study design during development. EC and RDB led the drafting of the work and all authors were involved in revising it. All authors gave final approval for publishing and agree to be accountable for what is published.

## DATA AVAILABILITY

All of the drug–drug interaction algorithms used in this study are provided in the supplement to this article. The data underlying this article cannot be shared publicly because access requires a data use agreement and approval from an institutional review board. The data will be shared on reasonable request to the corresponding author following approval from the institution that owns the data and institutional review board approval.

## FUNDING

This research was supported by the Agency for Healthcare Research and Quality (AHRQ) grant numbers R21 HS023826 and R01 HS025984 and National Science Foundation grant number 1838745.

*Conflict of interest statement*. None declared.

## Supplementary Material

ooab023_Supplementary_Data
