# Supplementary material for: Designing and evaluating contextualized drug–drug interaction algorithms
Source: JAMIA Open. 2021 Mar 19;4(1):ooab023. doi: 10.1093/jamiaopen/ooab023 (PMC7976224; doi:10.1093/jamiaopen/ooab023)
Supplement: ooab023_Supplementary_Data [file ooab023_supplementary_data.zip › Supplemental Figures.docx]

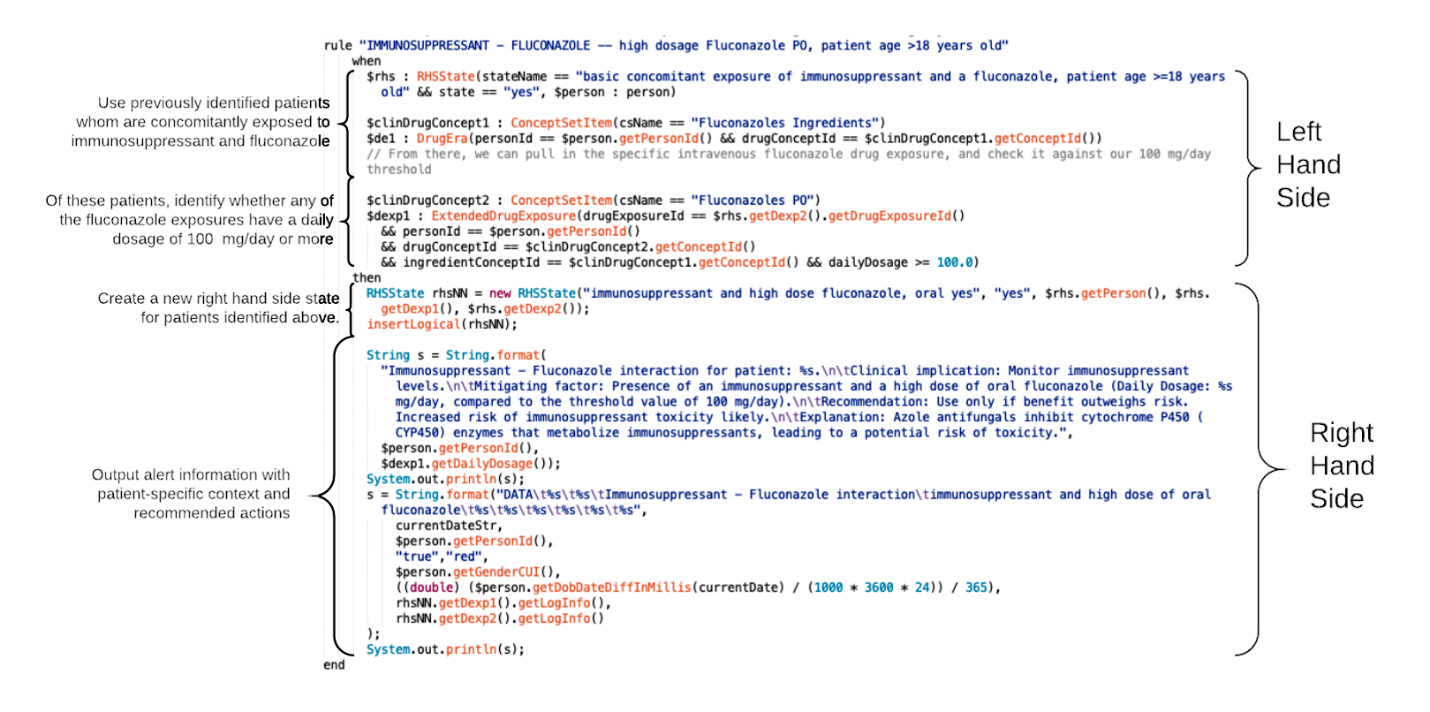


**Supplemental Figure 1.** A Drools rule for a single branch of the contextualized algorithm for “Immunosuppressant / Fluconazole” drug-drug interaction decision support. For each rule, the “Left Hand Side” references a previous decision point in the algorithm decision tree, applying additional filters on the data inherited from the prior decision point. The “Right Hand Side” of the rule uses the patients filtered from the “Left Hand Side” to create state object that can be referenced by other rules, as well as potentially output patient-specific alert information if the current rule is a leaf node in the decision tree.

#
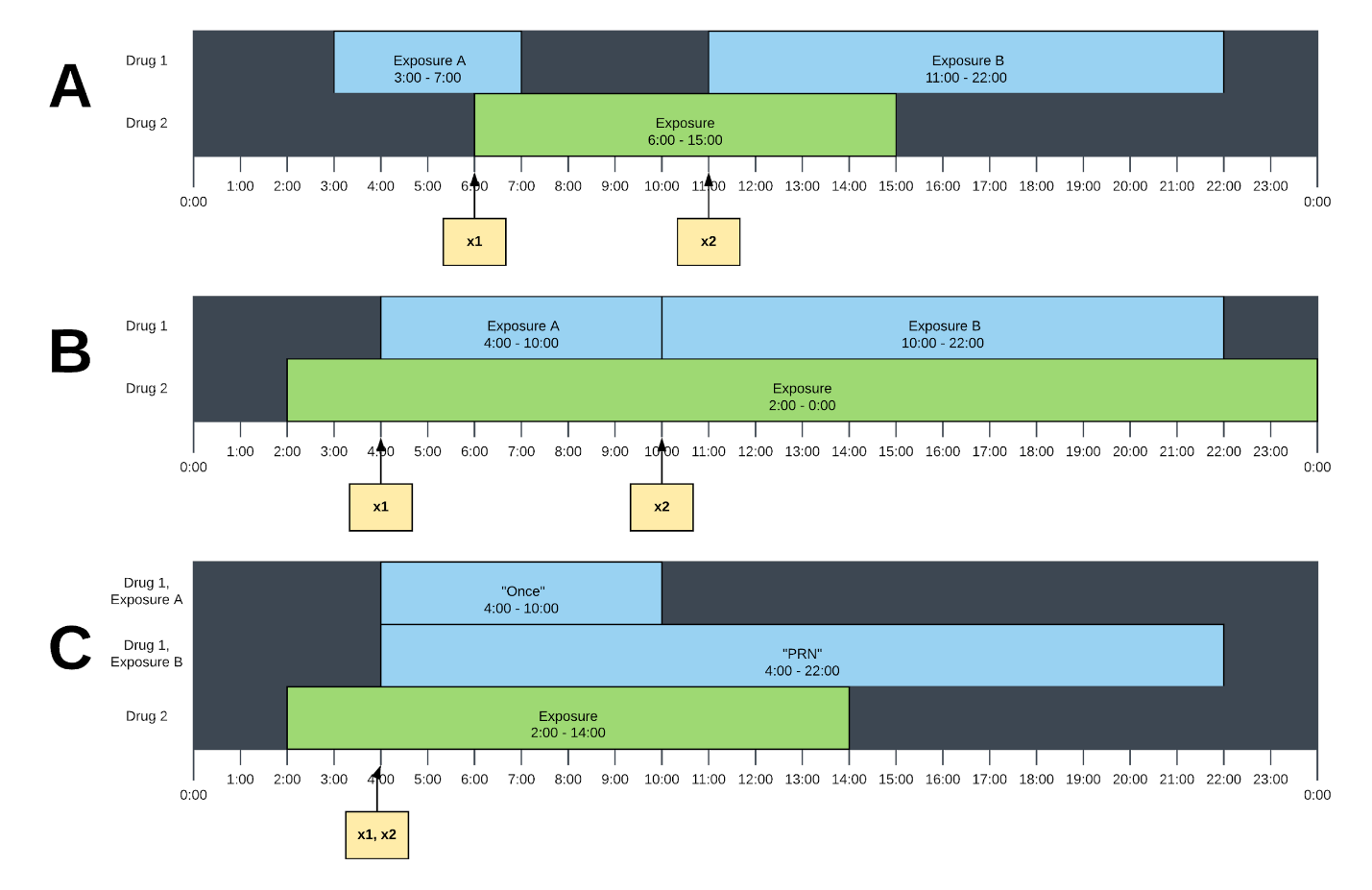


# Supplemental Figure 2.

Three timelines illustrating three theoretical scenarios of how basic concomitant exposures to drugs of interest were identified. The X-axis of each panel represents the 24 hours of a single day. Panels (A) and (B) illustrate two basic concomitant exposure events in the same day for Drug 1 and Drug 2, where Drug 1 has two distinct orders for the same patient in which the start times (x1 and x2) are different. Panel (C) also illustrates two basic concomitant exposure events in the same day for Drug 1 and Drug 2, except Drug 1 now has two distinct orders with the same start times (x1 and x2). Note that in panel C, although they share the same start times, the dosing information for each of the two distinct Drug 1 orders is different. Every unique exposure to the potentially interacting drugs indicated by an algorithm was counted. Cases where multiple drug exposures were started for the same drug at the exact same timestamp, but with different directions for dosage, were counted separately in the output. For instance, if one order for drug x had the dosage directions of “Once”, and different order for drug x had the same timestamp as the first order but with the dosage directions “PRN”, these were counted as two basic concomitant exposures. The decision to count each of these distinct drug exposures separately for the counts was based on the likely practical scenario that these orders were indeed entered separately in the clinical setting, and thus would hypothetically yield separate alerts for each of these medication orders.
