# Supplementary material for: Designing and evaluating contextualized drug–drug interaction algorithms
Source: JAMIA Open. 2021 Mar 19;4(1):ooab023. doi: 10.1093/jamiaopen/ooab023 (PMC7976224; doi:10.1093/jamiaopen/ooab023)

Alert only if you **stop** clonidine

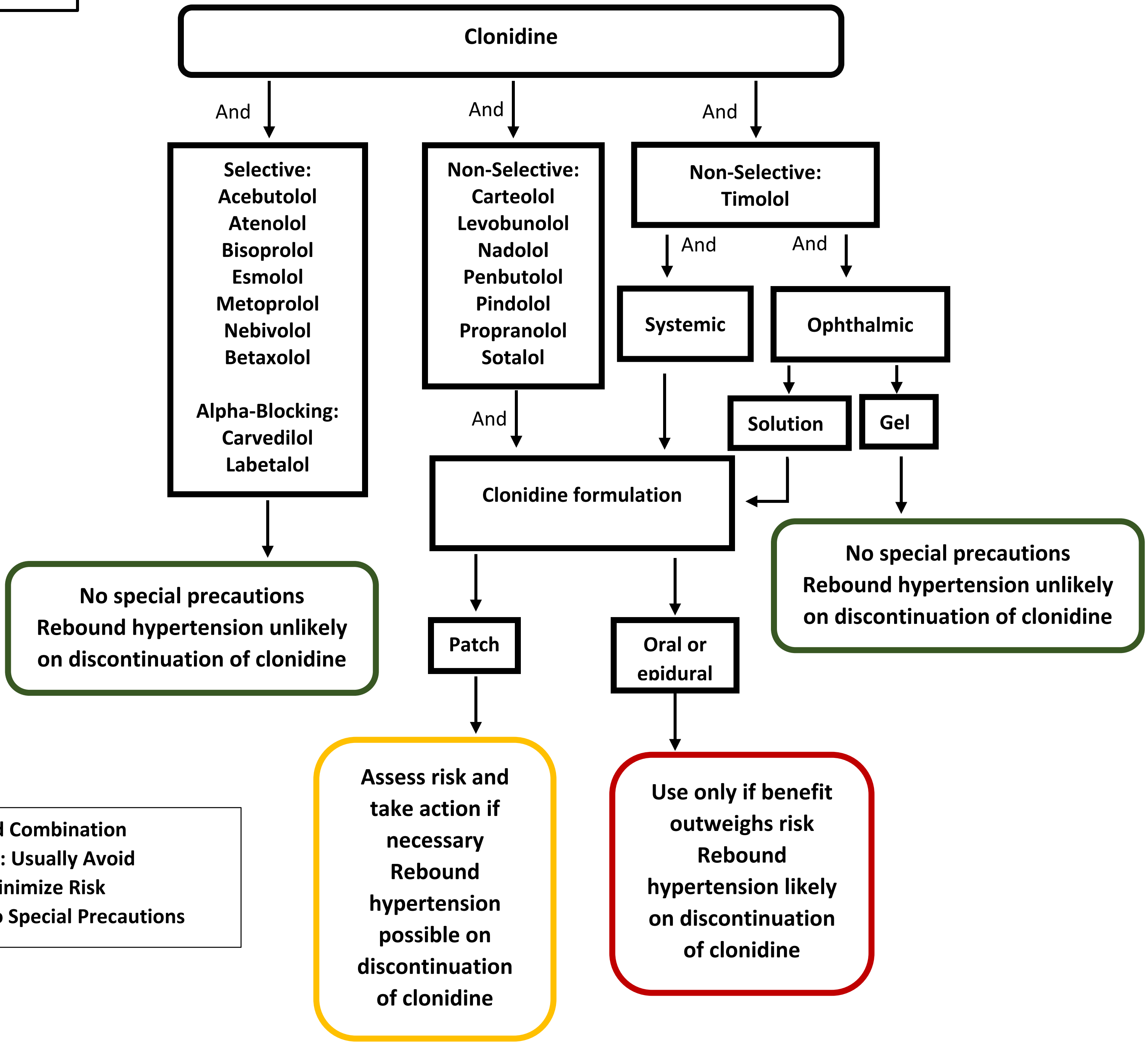

RED: Class 1: Avoid Combination
YELLOW: Class 2/3: Usually Avoid Combination or Minimize Risk
GREEN: Class 4: No Special Precautions

Beta-Adrenergic Blockers and Epinephrine

RED: Class 1: Avoid Combination
Yellow: Class 2/3: Usually Avoid Combination or Minimize Risk
Green: Class 4: No Special Precautions

mg = milligram

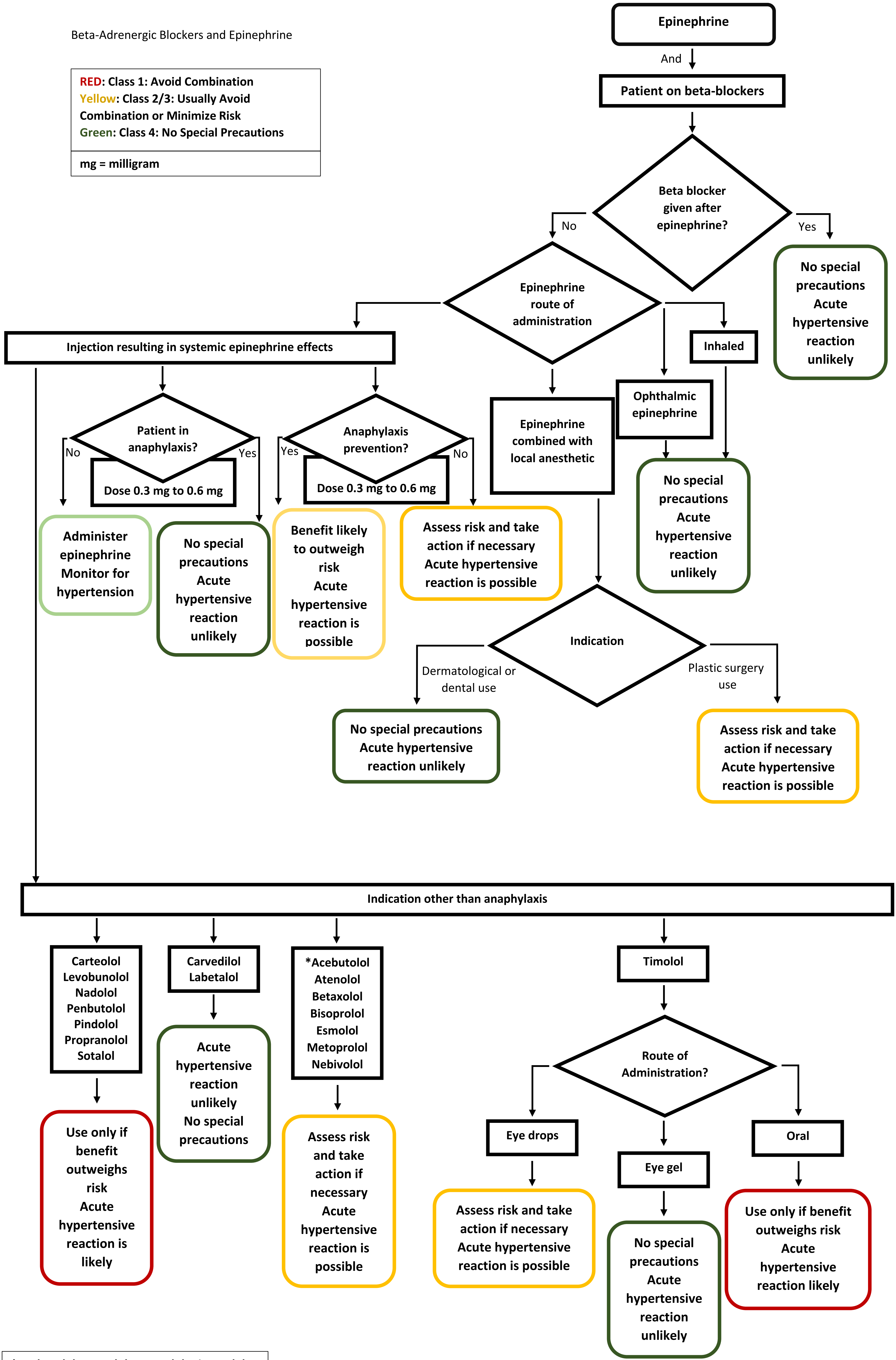

*Acebutolol, Atenolol, Betaxolol, Bisoprolol, Esmolol, Metoprolol, Nebivolol can become non-selective in higher doses.

Opioids and Fluconazole

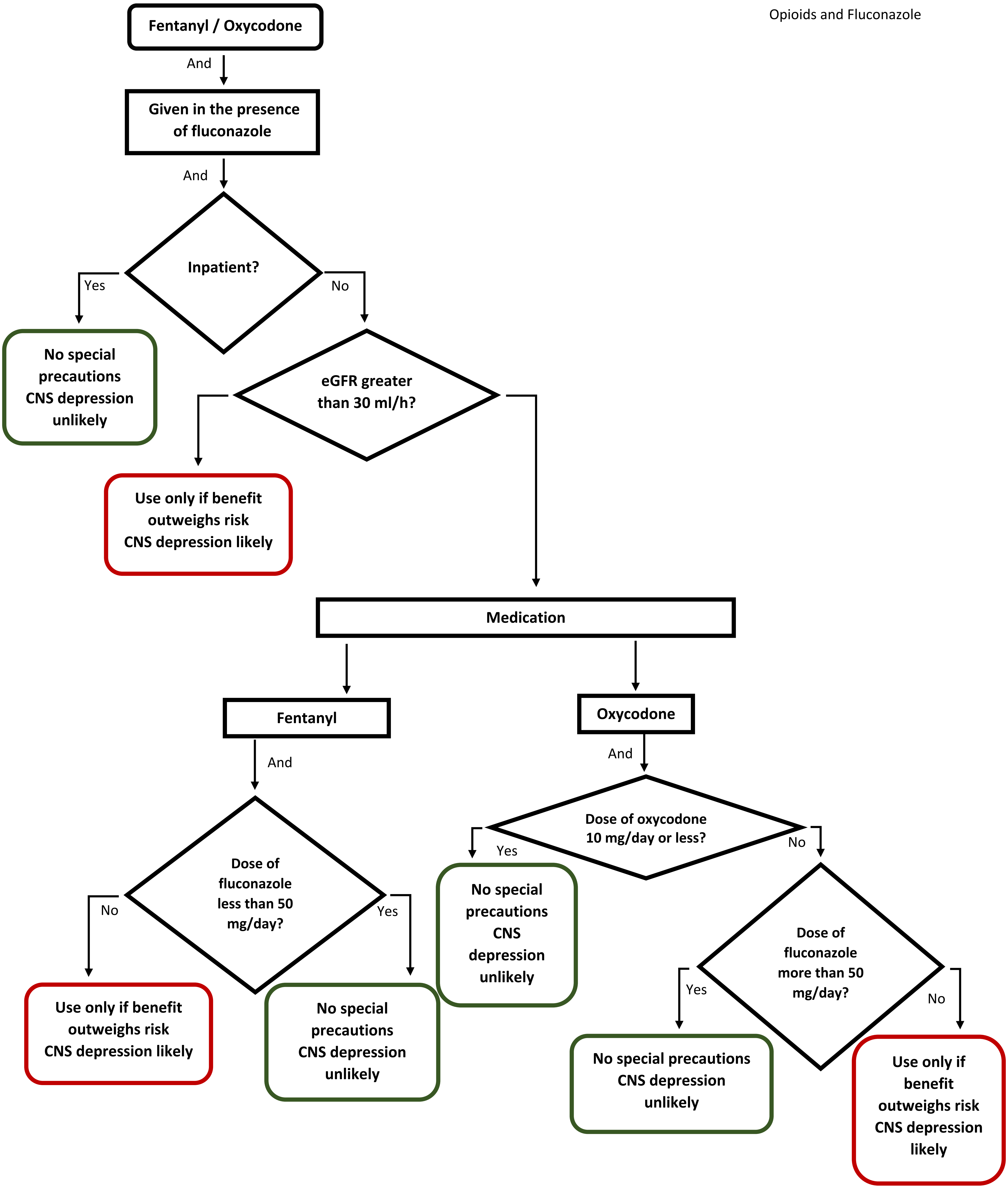

RED: Class 1: Avoid Combination
YELLOW: Class 2/3: Usually Avoid Combination or Minimize Risk
GREEN: Class 4: No Special Precautions

eGFR: estimated glomerular filtration rate
L = liter
ml = milliliters
CNS = central nervous system

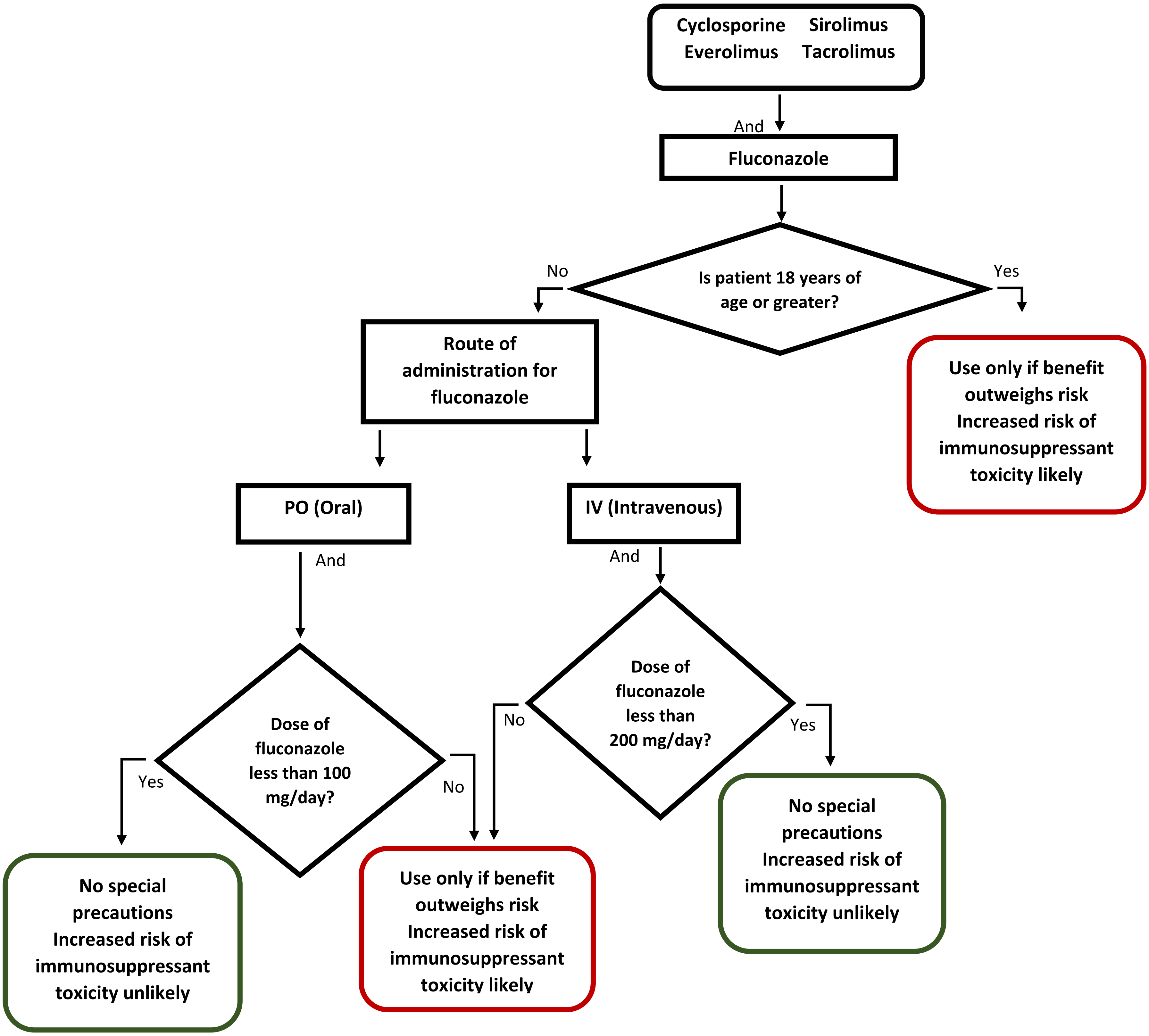

RED: Class 1: Avoid Combination
YELLOW: Class 2/3: Usually Avoid Combination or Minimize Risk
GREEN: Class 4: No Special Precautions

mg = milligrams

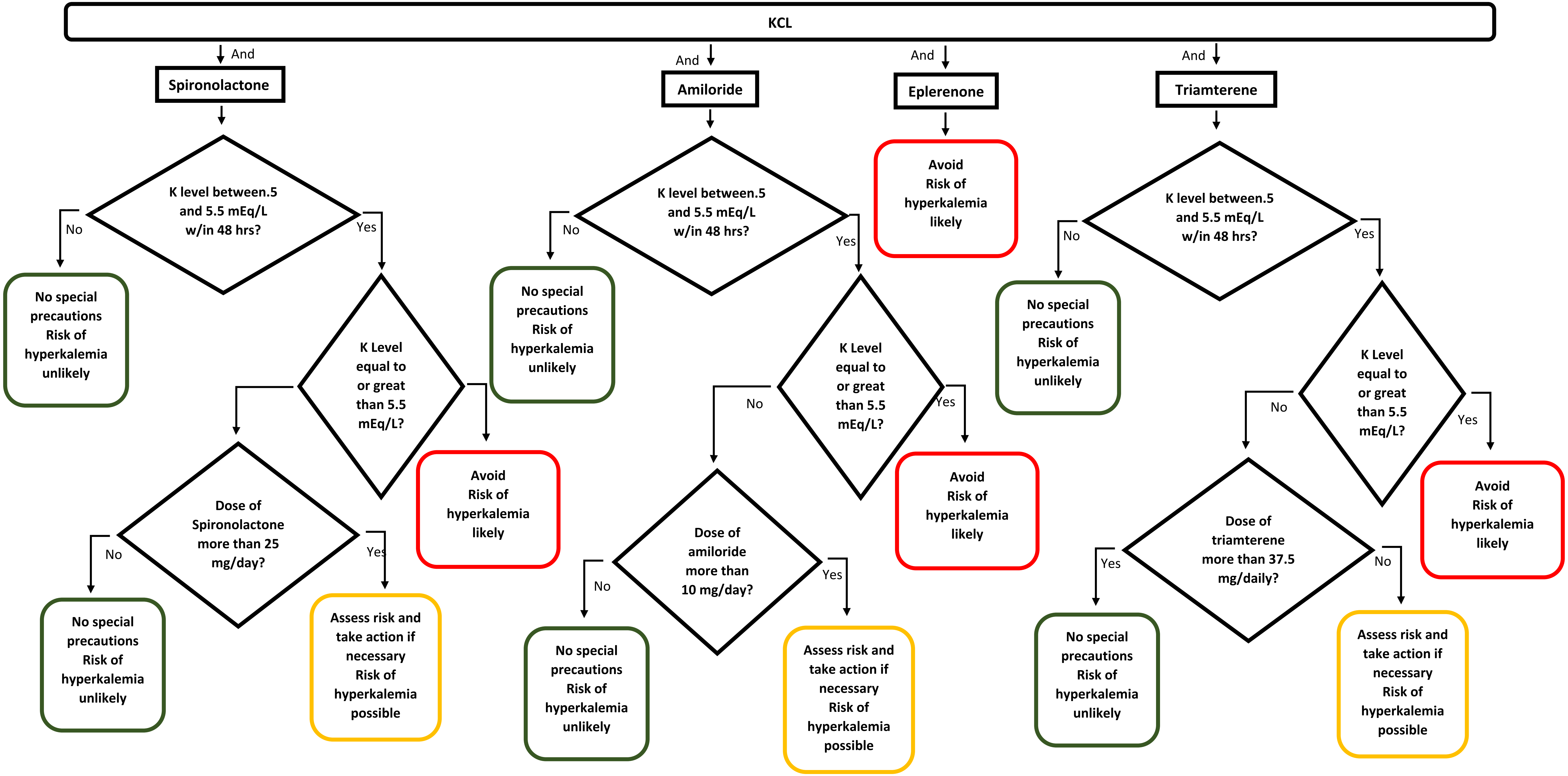

RED: Class 1: Avoid Combination
YELLOW: Class 2/3: Usually Avoid Combination or Minimize Risk
GREEN: Class 4: No Special Precautions

KCL = potassium chloride
K = potassium
hrs = hours
mEq = millieqvuivalents
L = liters
mg = milligrams

Warfarin and Antidepressants

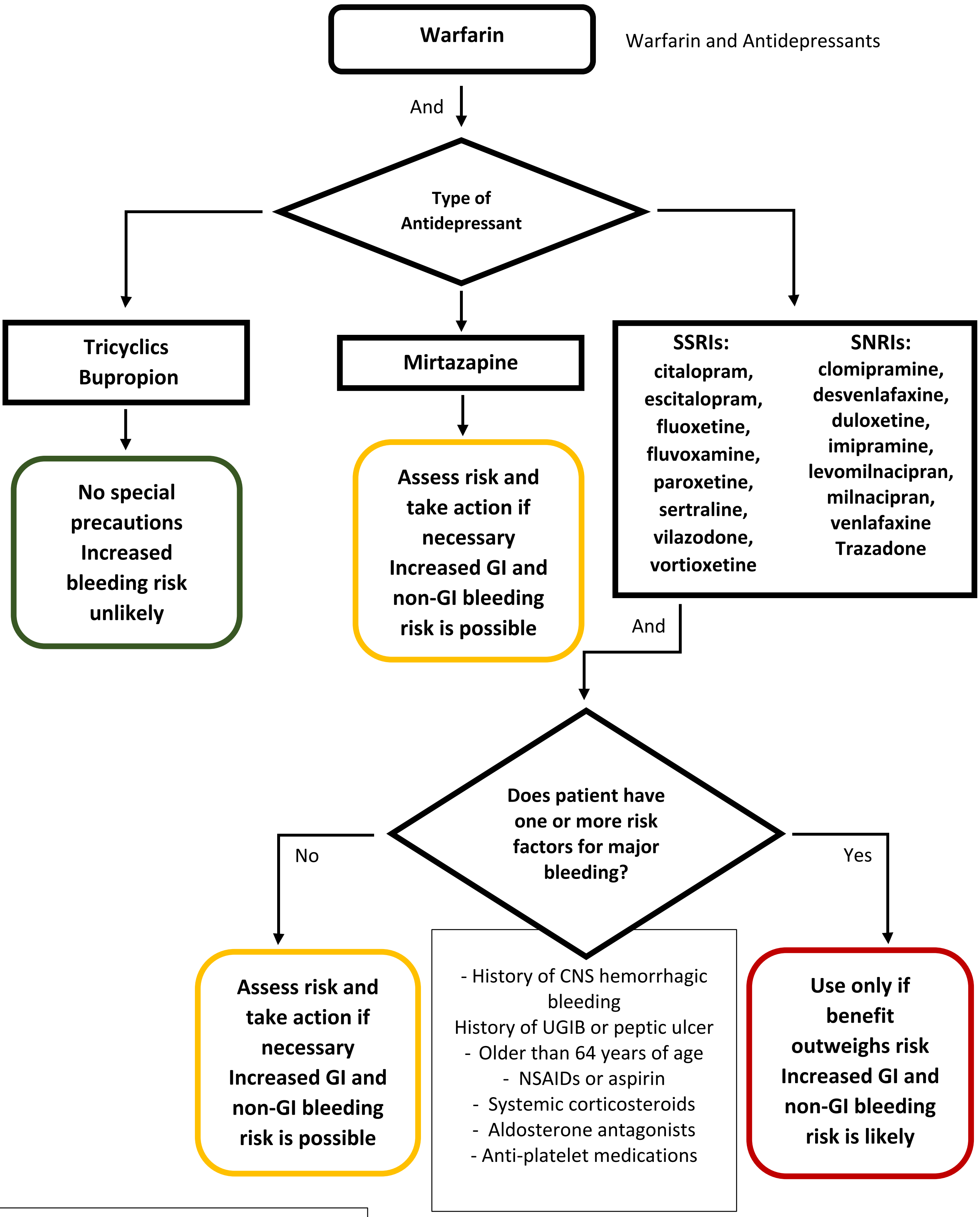

RED: Class 1: Avoid Combination
YELLOW: Class 2/3: Usually Avoid Combination or Minimize Risk
GREEN: Class 4: No Special Precautions

GI = gastrointestinal
CNS= central nervous system
NSAID= non-steroidal anti-inflammatory drugs

Warfarin and Salicylates

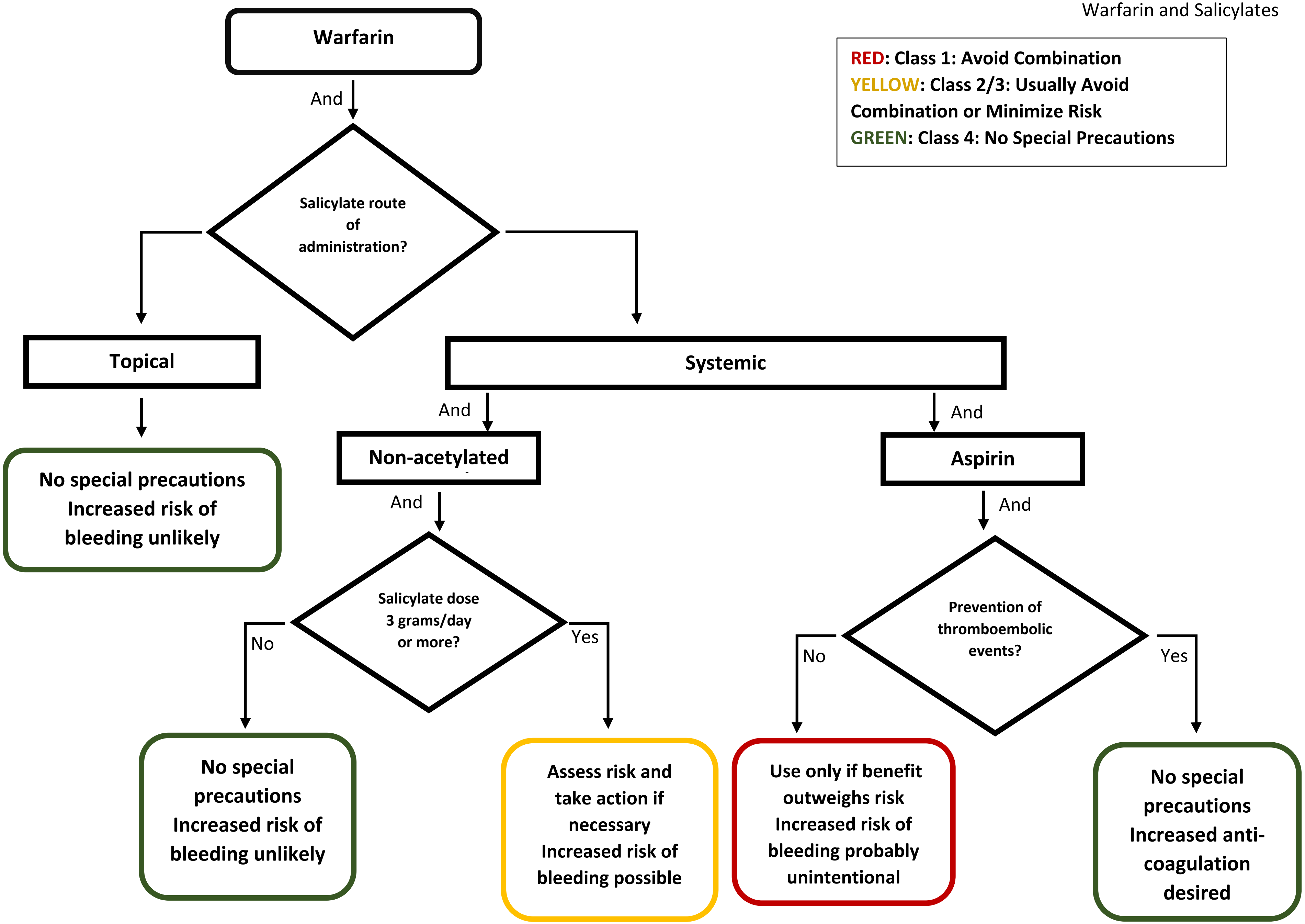

Citalopram and QT Prolonging Agents

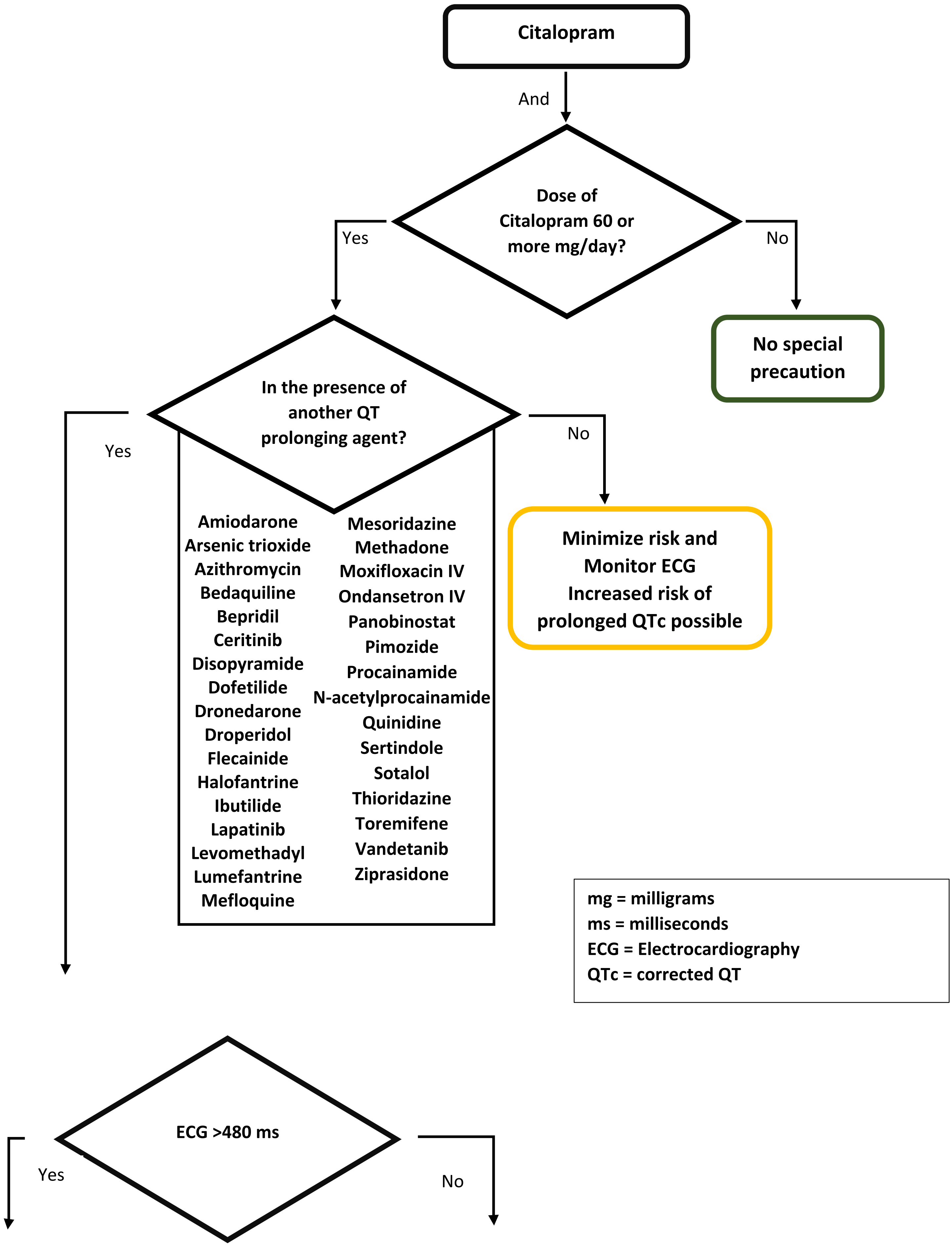

Citalopram and QT Prolonging Agents

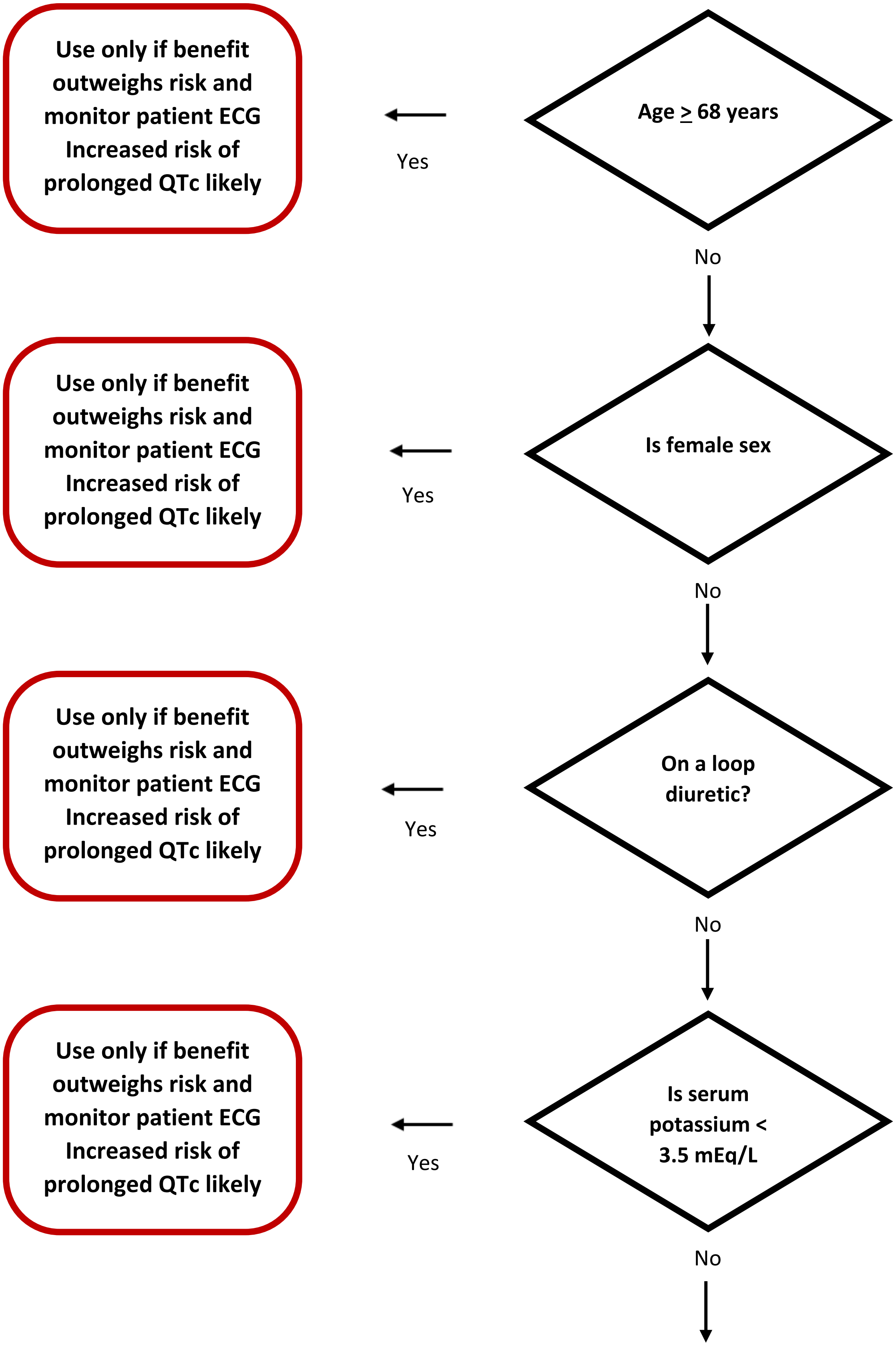

Citalopram and QT Prolonging Agents

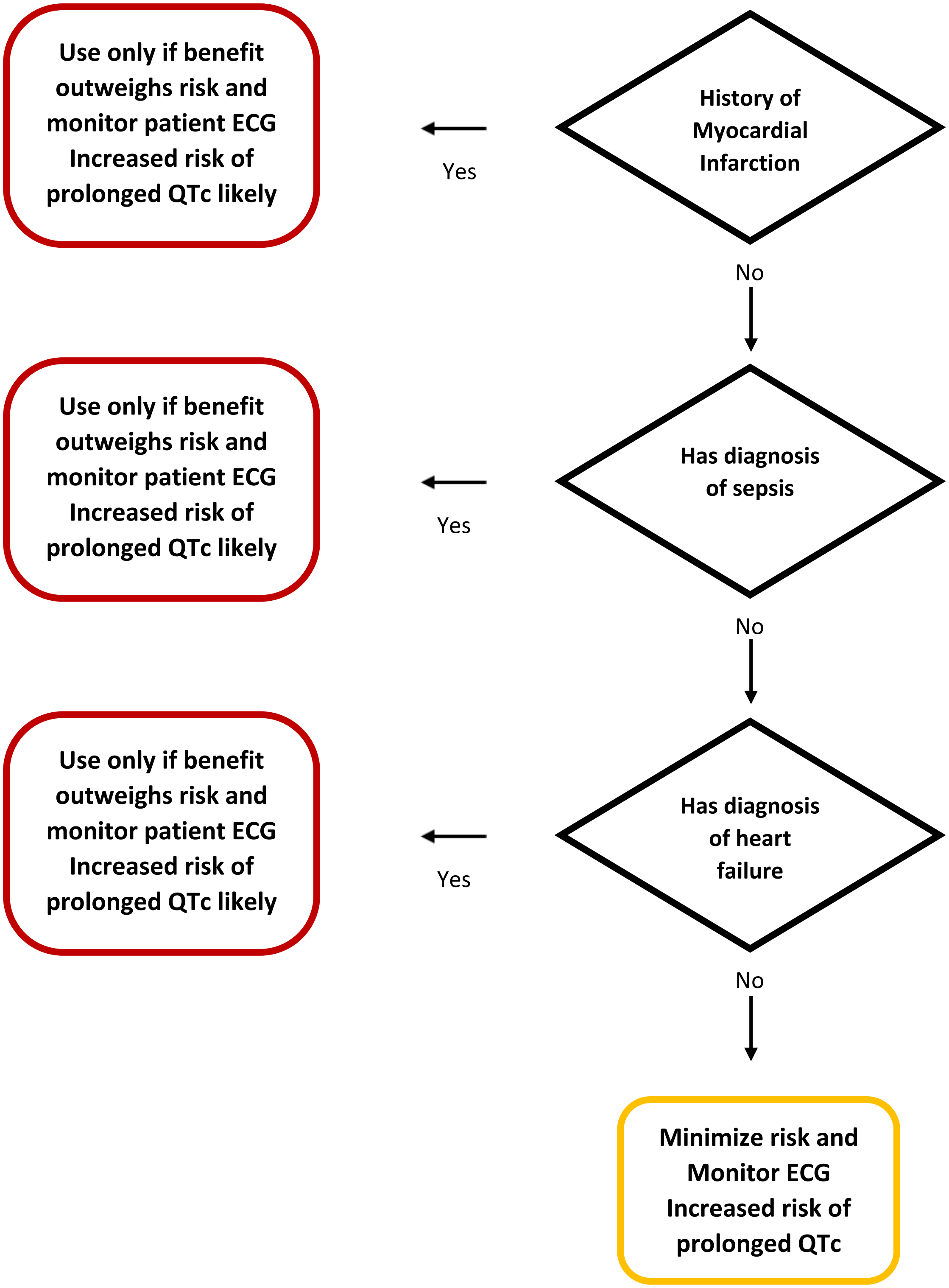

Supplement: ooab023_Supplementary_Data [file ooab023_supplementary_data.zip › supplemental-file-PDDI-algorithms.pdf]
